# Supplementary material for: Neural oscillations and top-down connectivity are modulated by object-scene congruency
Source: Cereb Cortex. 2025 Oct 21;35(10):bhaf290. doi: 10.1093/cercor/bhaf290 (PMC12539567; doi:10.1093/cercor/bhaf290)
Supplement: Gu_et_al_supp_materials_bhaf290 [file gu_et_al_supp_materials_bhaf290.docx]

**Supplementary materials for:**

**Neural oscillations and top-down connectivity are modulated by object-scene congruency**

Ye Gu^1^, Alexandra Krugliak^2^ & Alex Clarke^1,3,*^

^1^ Department of Psychology, University of Cambridge, UK

^2^ Cognition and Brain Sciences Unit, University of Cambridge, UK

^3^ Department of Psychology, University of Warwick, UK

**Corresponding author:**

Alex Clarke; [alex.clarke.3@warwick.ac.uk](mailto:alex.clarke.3@warwick.ac.uk); Department of Psychology, University of Warwick. CV4 7AL, UK.

**Supplementary Figure 1**


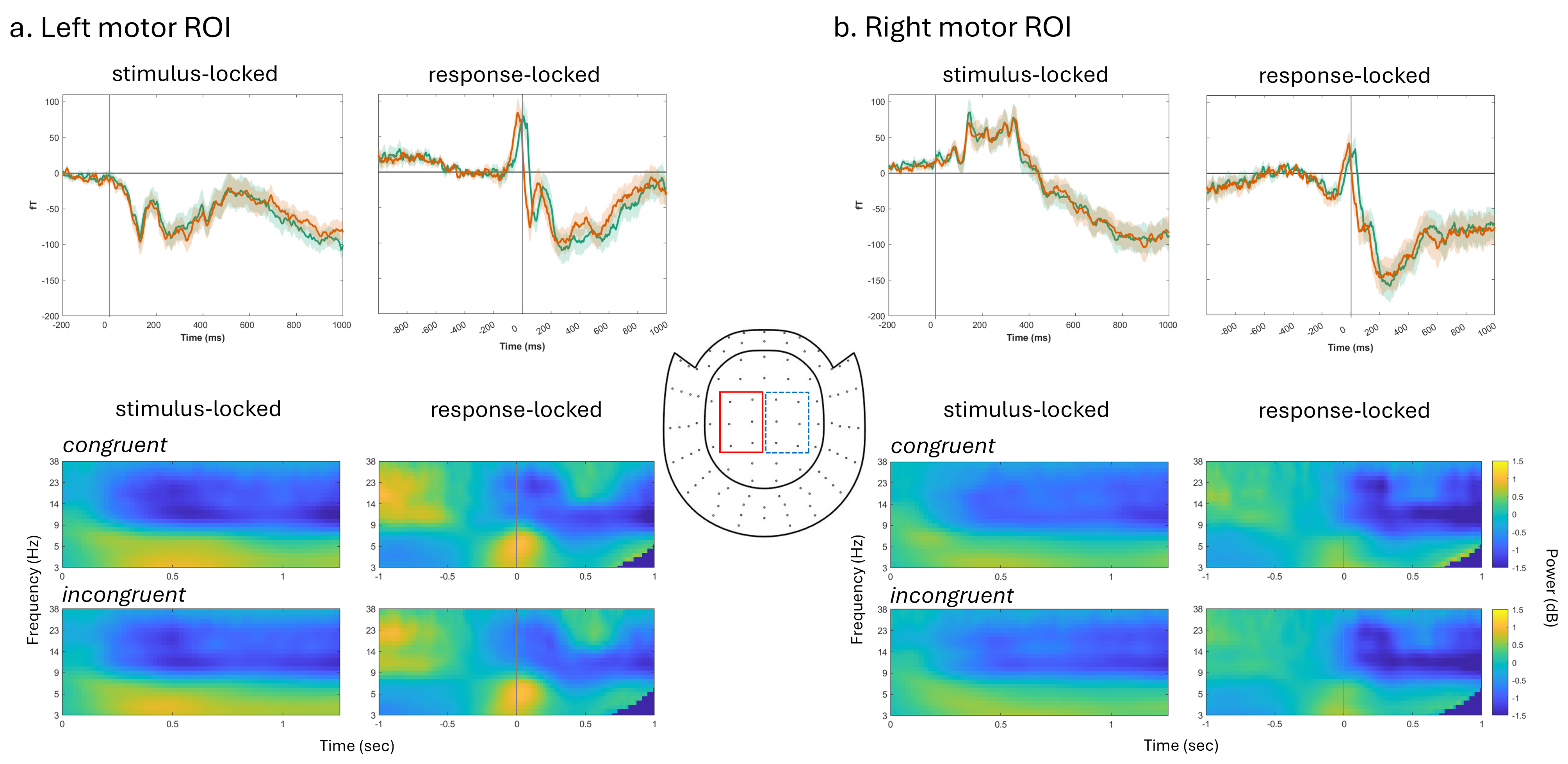


Supplementary figure 1. Control motor analysis. To rule out that our effects could reflect motor preparation, we performed an additional analysis targeting sensors above the (a) left and (b) right motor cortex. Both stimulus-locked and response-locked analyses across the combined motor region showed no significant differences between congruent (green) and incongruent (orange) trials, in either the event-related data (top row) or the time frequency representations (bottom row). The central topographic image shows the sensors the left and right motor regions were plotted from.
